# Loss of both diffusion restriction and arterial enhancement can predict complete pathological response following Y-90 selective internal radiation therapy for hepatocellular carcinoma

**DOI:** 10.1007/s00261-026-05377-5

**Published:** 2026-02-09

**Authors:** Kah Heng Alexander Lim, Matthew Joel Clifford, William Ormiston, Tze Sheng Khor, Khanh Quoc Steven Ngo, Alistair Rowcroft, Lingjun Mou, Luc Delriviere, Michael Wallace, Mayank Bhandari, Mohammad Ballal, Jonathan Tibballs

**Affiliations:** 1 Sir Charles Gairdner Hospital, Nedlands, Australia; 2 University of Western Australia, Perth, Australia; 3 Pathwest Laboratory Medicine, Nedlands, Australia; 4 Sir Charles Gairdner Hospital, Nedlands, Australia; 5 Fiona Stanley Hospital, Perth, Australia; 6 Curtin University, Perth, Australia

**Keywords:** Selective internal radiation therapy (SIRT), Hepatocellular carcinoma (HCC), mRECIST, Diffusion weighted imaging (DWI), Diffusion restriction, Pathological response

## Abstract

**Purpose:**

To assess the association of diffusion restriction on diffusion weighted MRI (DWI) with complete pathological necrosis (CPN) in hepatocellular carcinoma (HCC) following selective internal radiation therapy with Yttrium-90 resin microspheres (SIRT).

**Methods:**

A retrospective cohort study of patients undergoing resection or transplantation for HCC following SIRT was performed. Imaging pre- and post-SIRT was assessed for response to treatment via mRECIST and for the presence of diffusion restriction on DWI. Histological specimens were assessed for complete pathological necrosis (CPN).

**Results:**

Twenty-nine tumours were included from 25 patients; mRECIST complete response (CR) demonstrated moderate reliability (sensitivity 0.92, specificity 0.68) for predicting CPN. Twelve tumours did not have diffusion restriction on pretreatment DWI; following their exclusion, loss of diffusion restriction demonstrated a weak agreement (sensitivity 0.85, specificity 0.80, Cohen’s Kappa 0.197) for prediction of CPN. The combination of mRECIST CR and loss of diffusion restriction was associated with CPN in 100% (6/6) cases; whilst persistent abnormal diffusion restriction despite mRECIST CR was associated with residual disease in 75% (3/4) cases.

**Conclusion:**

The combination of mRECIST CR when combined with loss of diffusion restriction appears to predict CPN post SIRT; persistent diffusion restriction despite the findings of mRECIST CR appears to correlate with residual disease.

## Introduction

Selective internal radiation therapy (SIRT) using Yttrium-90 labelled microspheres has evolved to becoming an invaluable component of the multidisciplinary management of hepatocellular carcinoma (HCC). Where evidence of residual disease is present on post-treatment imaging, patients who are surgical candidates may progress to resection or transplantation [1–3]. however radiological assessment of response following SIRT is challenging due to necrosis of tumour and radiation-induced fibrosis and altered post-contrast enhancement patterns of surrounding parenchyma that confounds volume based assessments.

Size change alone is unreliable for assessment of treatment response in HCC post SIRT [4]; leading to the popularity of enhancement based response criteria such as mRECIST and EASL in clinical and research settings [1, 5–7] with further refinement towards the concept of ‘mass like’ enhancement in the Li-RADS-TR algorithm [8]. The gold standard for determination of complete pathological necrosis (CPN) and residual disease remains histopathological evaluation of the surgical specimen; however surgery post SIRT is challenging, carries significant morbidity [9, 10], and may be unnecessary in cases where treatment with SIRT has been definitive and CPN of a target lesion is obtained [11].

Diffusion-weighted magnetic resonance imaging (DWI) allows assessment of changes in intratumoral water diffusion which can manifest as early as 40 days post SIRT [12]. Assessment of diffusion restriction is performed either by radiologist’s qualitative interpretation of signal intensity on diffusion weighted sequences, or quantitatively through the measurements of apparent diffusion coefficients (ADC); the latter itself requiring manual and arguably subjective segmentation of the treated tumour from surrounding irradiated parenchyma where demarcation may be lost [13].

While previously established tools for assessing treatment response such as mRECIST and EASL do not consider the role of DWI in residual tumour, the 2024 LiRADS Treatment Response Algorithm introduces diffusion restriction of any degree as an ancillary feature that can be applied optionally at the user’s discretion to favour conversion of non-progressive to viable disease [8]. There remains however limited radiological-pathological data to validate the use of DWI in treatment response assessment.

The aim of this study was to evaluate the diagnostic value of loss of diffusion restriction assessed qualitatively on DWI as a complement to mRECIST for the prediction of CPN in a population of HCC treated with SIRT, followed by resection or transplantation.

## Methods

### Study design

A retrospective cohort study of patients with HCC treated with SIRT followed by either liver resection or transplantation from two tertiary centres in Western Australia.

Participant inclusion/exclusion criteria: A search of institutional liver transplant, interventional radiology and anatomical pathology databases were performed to identify patients from January 2010 to July 2024. Criteria for inclusion was a patient with radiologically or biopsy diagnosed HCC and previous SIRT treatment followed by either surgical resection or liver transplantation, that had at least one pre-operative MRI scan to assess for DWI. For patients with multiple tumours, radiological and macroscopic photographic correlation was performed to ensure correct lesion assignment, which was limited to the two largest lesions. For patients that underwent previous non-SIRT locoregional treatment (specifically ablative treatments), only the tumour(s) treated with SIRT were included for analysis; tumours that underwent SIRT but also fell within the treatment zone of previous non-SIRT therapies were excluded.

### Radiological assessment

At our study institutions, patients with HCC tumours treated with SIRT undergo routine imaging with contrast enhanced multi-phase CT and MRI with the majority of patients undergoing the following MRI protocol: Coronal and axial T2 HASTE, T1 in and out of phase, T2 BLADE axial, T1 VIBE axial and coronal fat sat non-contrast and dynamic post contrast studies, and DWI (B50, B400, B800 and Apparent Diffusion Coefficient [ADC] map).

Pre and post-SIRT imaging studies were retrieved and read by a consultant interventional radiologist (MC) with indeterminate cases discussed with a second consultant interventional radiologist for a consensus read. Lesion size was taken as the maximal arterial enhancement diameter pre-SIRT.

**Fig. 1:**
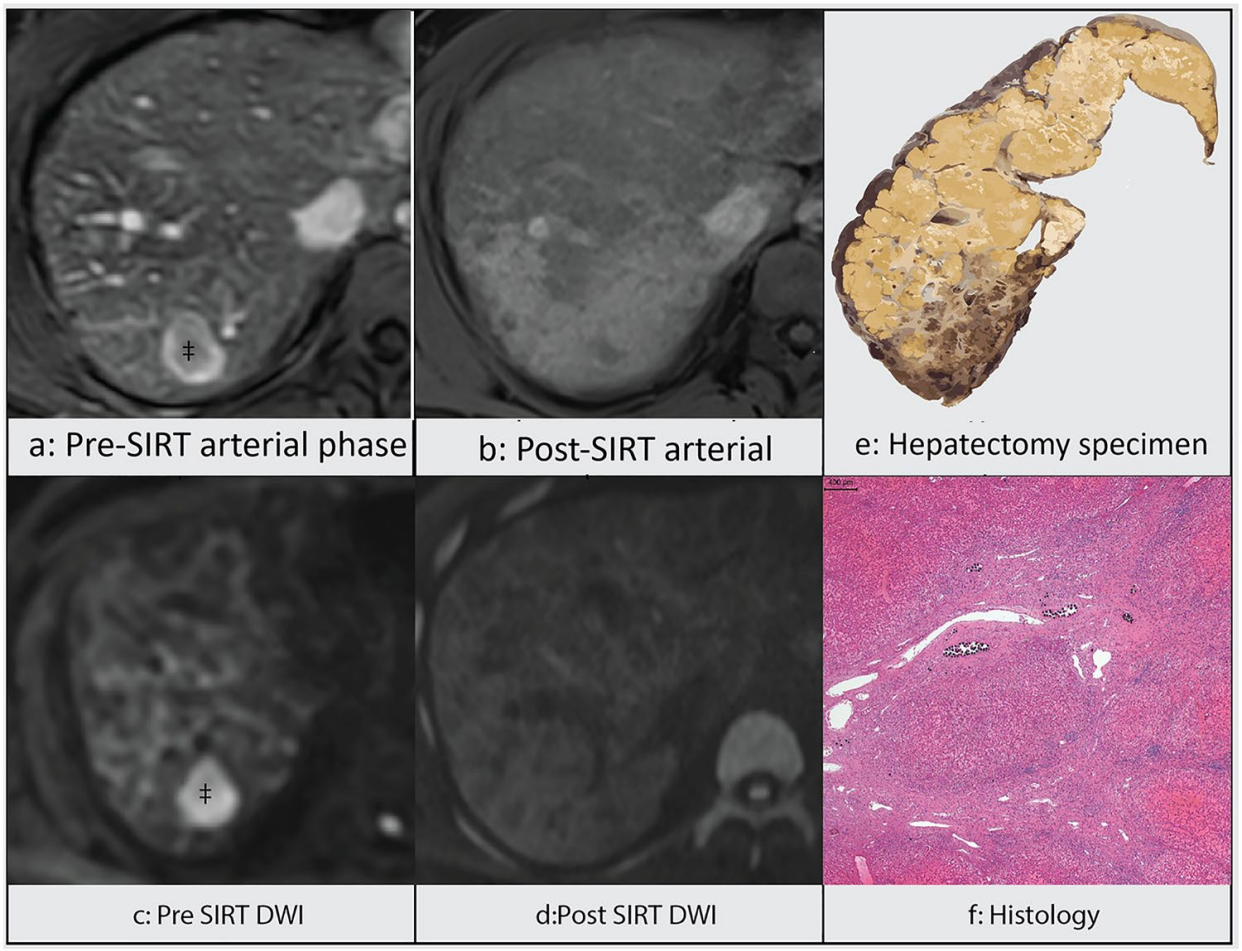
**a**–**f**: Tumour (‡) demonstrating loss of both arterial enhancement (**b**) and diffusion restriction (**d**) post SIRT with tumour demonstrating CPN (**f**) on histopathology

**Fig. 2:**
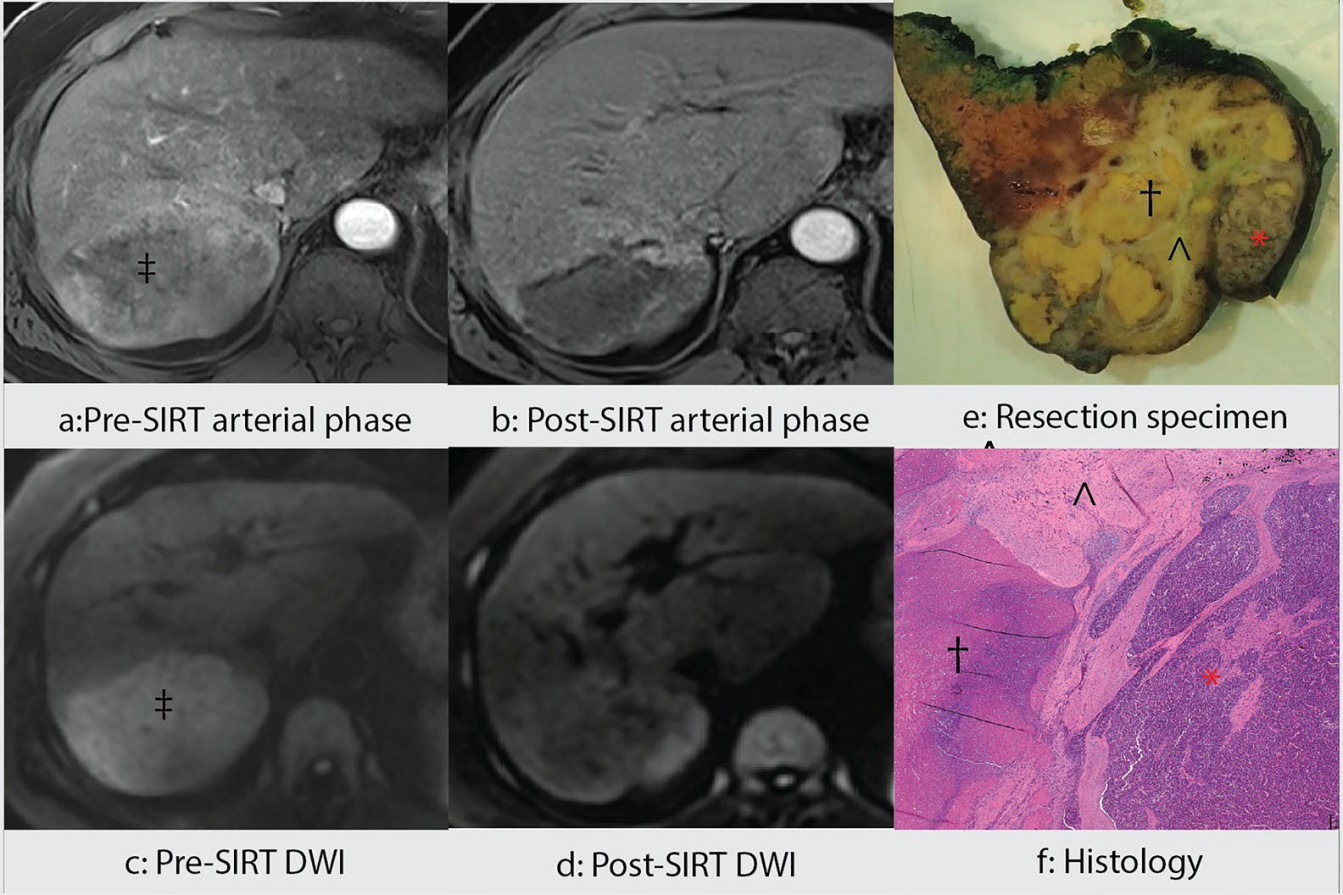
**a**–**f** Tumour (‡) demonstrating loss of arterial enhancement post SIRT (**b**) but persistent abnormal signal on DWI medially (**d**); pathological assessment of this region identified focus of residual tumour (*) interspersed with fibrosis (^) and tumour necrosis (†)

Post treatment tumours were classed using mRECIST criteria [14]. Pre-treatment and post treatment DWI lesional signal relative to background liver was assessed; tumours were categorised qualitatively as having either complete loss of diffusion restriction (Fig. 1c, d) or persistent abnormal signal (Fig. 2c, d) post-SIRT, or having no diffusion restriction at baseline i.e. pre-SIRT. Heavily T2 weighted sequences and T1 weighted sequences were used to identify potential sources of error in DWI assessment such as T2 shine through and hemorrhage. For example, if T2 hyperintense material which appeared consistent with fluid was observed along with a subjective high signal ADC map, the conclusion would be made that there was no true diffusion restriction.

### Histopathology assessment

Macroscopic photos of resections or explanted livers were assessed and slides retrieved for histological scoring by a consultant pathologist specialising in liver pathology (TK). Pathological response were scored as complete pathological necrosis (CPN; 0% viable tumour cells), followed by < 5%, 5–10%, 10–20%, 20–50%, and > 50% residual viable tumour.

### Statistical analysis

Median and interquartile ranges (IQR) were presented for continuous variables and differences assessed using Mann Whitney U test with a p value of < 0.05 for significance. For analysis, mRECIST scoring was binarized into complete response (CR) vs. no CR (partial response, stable or progressive disease categories); DWI was categorised into loss of diffusion restriction, persistent diffusion restriction, or no baseline diffusion restriction. Pathological assessment was similarly binarized to CPN or residual disease.

Performance of mRECIST or DWI in predicting pathologic complete necrosis was expressed in raw agreement, sensitivity and specificity. Strength of agreement was assessed using Cohen’s Kappa with adjustment for small sample size and imbalance of prevalence carried out using Prevalence-adjusted Bias-adjusted Kappa (PABAK); for both, values between 0.4 and 0.75 were used to indicate moderate to good agreement.

### Ethics

Approval for the study was obtained from the Human Research Ethics Committee of Sir Charles Gairdner Hospital (Project Record Number: RGS6872).

## Results

A total of 29 HCC tumours from 25 patients fulfilled criteria for inclusion; of whom 40% underwent transplant and 60% resection. Median age at time of SIRT was 62 years, with majority male (88%). All cases had the diagnosis of HCC made on radiological and clinical grounds in a multidisciplinary consensus meeting, with an additional 11 cases (44%) undergoing pre-treatment biopsy for confirmation. As expected from the inclusion criteria (i.e. candidates for SIRT and subsequent surgery) the majority of included patients had an ECOG of 0 (*n* = 20; 80%) and were either non-cirrhotic (*n* = 9, 36%) or had Child-Pugh A cirrhosis (*n* = 13, 52%). No patient received concomitant systemic immunotherapy or chemotherapy. The demographics, liver disease etiology, and prior treatment history for the cohort are expanded in Table 1.

**Table 1:** Demographics, etiology, and severity of liver disease for patient cohort (*n*=25)

	Median (IQR)/Frequency (%)
Demographic	
Age	61.76 (36.72–78.14)
Male gender	22 (88%)
Etiology	
Hepatitis B	4 (16%)
Hepatitis C	2 (8%)
Alcohol	2 (8%)
Viral/alcohol	7 (28%)
NAFLD	6 (25%)
Other/unknown	4 (16%)
Child-Pugh	
A	13 (52%)
B	3 (12%)
Non-cirrhotic	9 (36%)
ECOG performance status	
0	20 (80%)
1	5 (20%)
BCLC Stage	
0	1 (4%)
A	3 (12%)
B	15 (60%)
C	6 (24%)
MELD score	8.6 (6–15)

*ECOG* Eastern cooperative oncology group, *BCLC* Barcelona clinic liver cancer, *MELD* Model for end stage liver disease

Thirteen tumours (44.8%) demonstrated CPN on resection or explant pathological assessment; age, sex, and interval (in days) from SIRT to surgery were not associated with CPN. Lesion size (taken as maximum enhancement diameter pre-SIRT) was significantly smaller for complete response (51 mm [28.00–60.50.00.50] vs. incomplete response 65.5 mm [44.25–93.00.25.00] (*p* = 0.05). These findings are summarized in Table 2.

**Table 2:** Demographics, tumour size, and time interval from SIRT to surgery for residual disease vs. CPN groups

	Residual disease (*n*=16)	Complete pathologic necrosis (*n*=13)	*P* value
Age (years)	63.5 (58.25–68.25)	64 (46–74.5.5)	0.98 *
Sex	M=13 F=3	M=13	0.23 Ɨ
Max enhancement diameter pretreatment (mm)	65.5 (44.25–93.00.25.00)	51 (28.00–60.50.00.50)	0.050 *
Time from SIRT to surgery (days)	158 (141.5–387.25.5.25)	330 (166.5–392.5)	0.184 *

*Mann Whitney U; Ɨ Fishers exact test

**Fig. 3:**
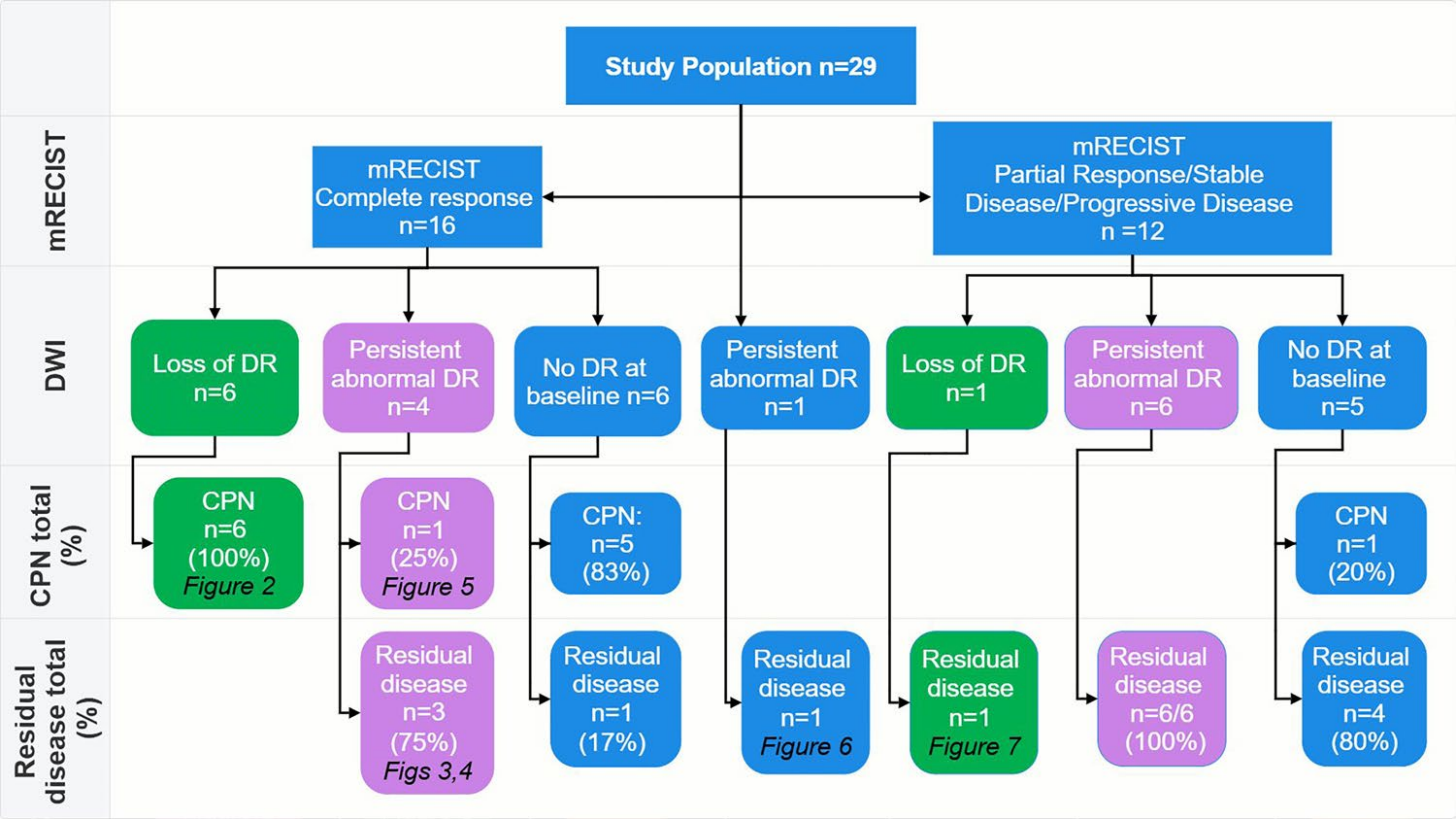
Flowchart depicting the relationship between mRECIST, DWI and pathological outcome

**Fig. 4:**
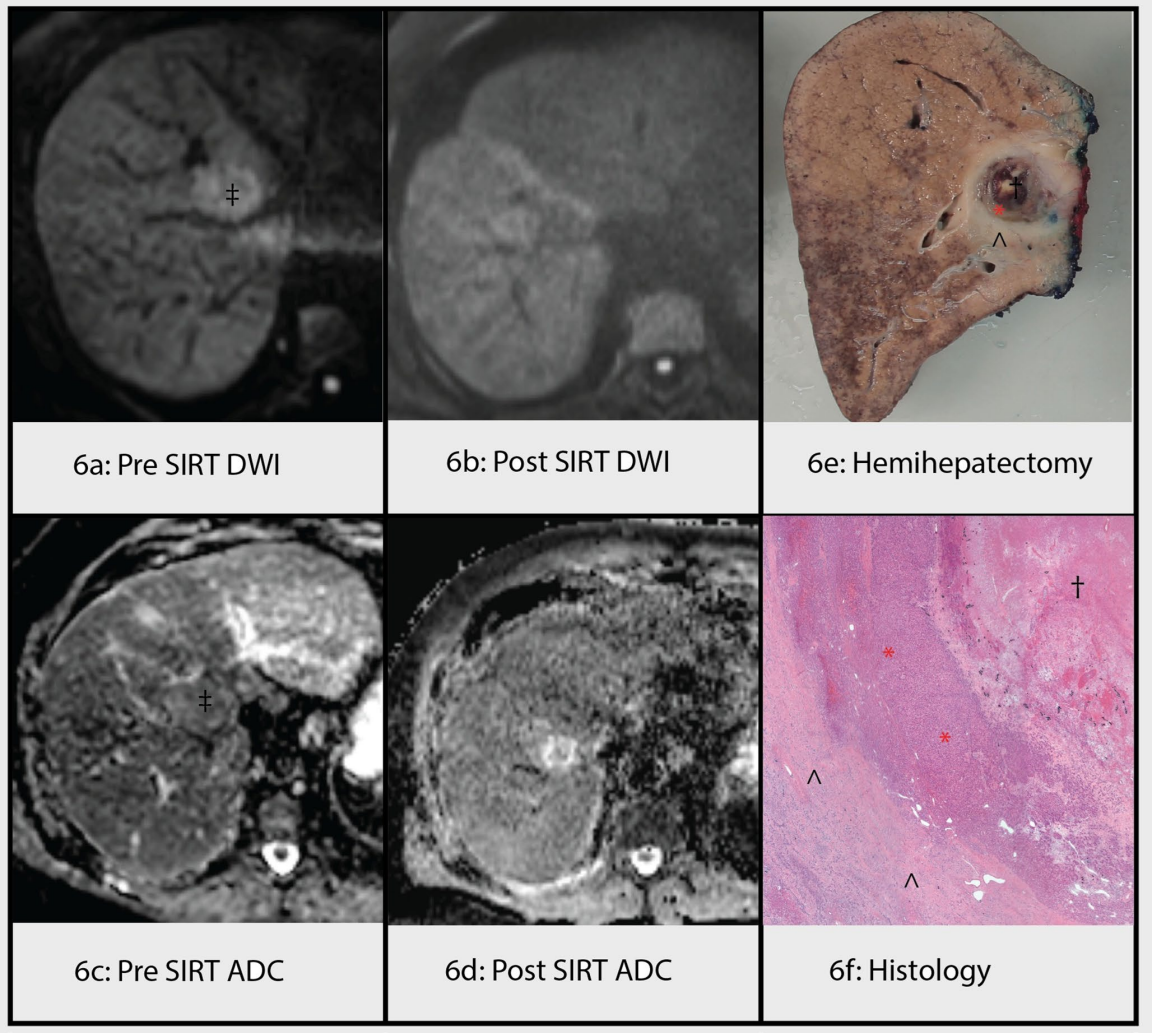
**a**–**f** Tumour (‡) demonstrating persistence of diffusion restriction on DWI and ADC map post SIRT (**a**–**d**; no arterial phase was available so mRECIST could not be assessed). Residual viable tumour (*) was present at the periphery (estimated 50%) of tumour with a necrotic core (†), and surrounding fibrosis (^)

One tumour did not have appropriate posttreatment contrast enhanced imaging for mRECIST scoring (Figs. 3, 4); excluding this case, the agreement between mRECIST and CPN appeared to be moderate (raw agreement = 0.79, sensitivity = 0.92, specificity = 0.68) with Cohen’s Kappa of 0.59 indicating that the modest agreement is beyond chance and PABAK of 0.58 indicating stability after adjusting for small sample size and imbalance in prevalence.

Eleven tumours did not demonstrate diffusion restriction prior to treatment and were excluded from further statistical analysis. This reduced the sample size to 17 tumours for calculation of agreement metrics for loss of diffusion restriction and complete pathologic response. While simple metrics of raw agreement = 0.82, sensitivity = 0.85, and specificity = 0.80 appear to outwardly suggest good agreement, the low Cohen’s Kappa = 0.197 indicates there is only a weak agreement beyond chance despite the more favourable PABAK = 0.647 which reflected class imbalance caused by small numbers; ultimately requiring cautious interpretation.

Regarding the association of time interval from SIRT to re-imaging; there was no significant difference in median [IQR] number of days from SIRT to follow up imaging for mRECIST response categories (CR = 177 [107–321] days; no CR = 163 [86–341] days, *p* = 0.65) or DWI categories (loss of diffusion restriction = 180 [103–230] days; persistent diffusion restriction = 127 [99–370] days, *p* = 0.70).

**Fig. 5:**
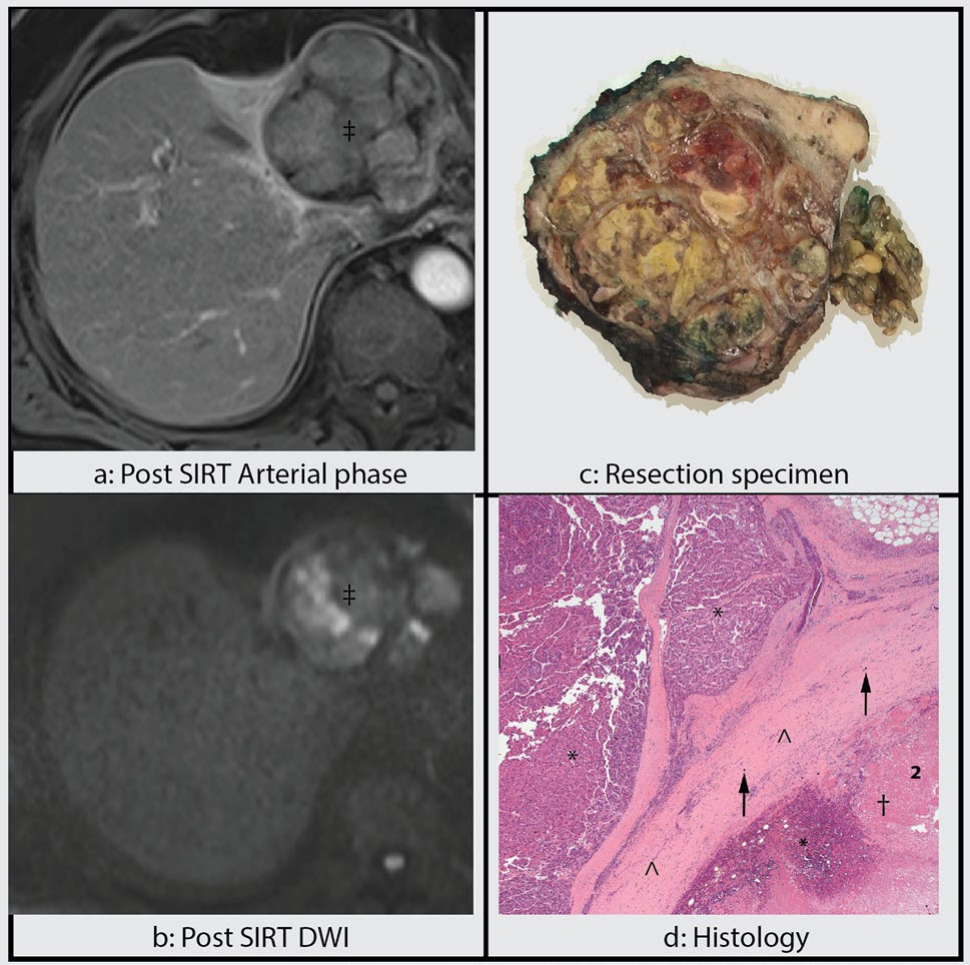
**a**–**d** Tumour (‡) demonstrating loss of arterial enhancement post SIRT (**a**) but persistent abnormal signal on DWI (**b**), histopathology revealing corresponding foci of residual viable tumour (*), regions of necrotic tumour with adjacent hemosiderin (†), bands of fibrosis (^), and SIRT beads (arrows)

**Fig. 6:**
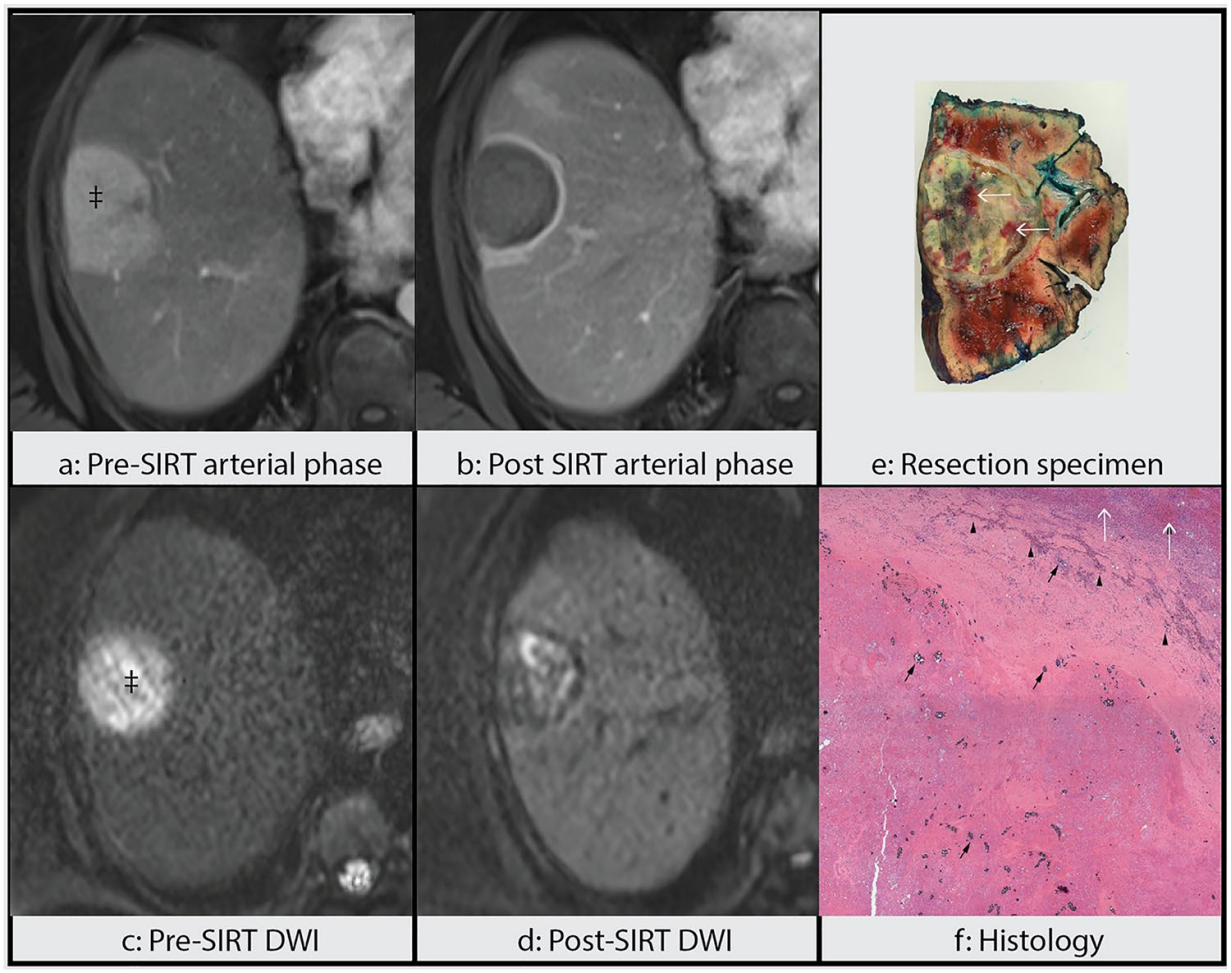
**a**–**f** Tumour (‡) demonstrating loss of arterial enhancement (**a**, **b**) but residual abnormal signal on DWI (5**c**, 4 **d**) corresponding to regions of intratumoral hemorrhage (white arrows 4e and 4f) in otherwise complete necrotic tumour with SIRT beads (short arrows) and hemosiderin depositation (arrowheads) (**f**)

**Fig. 7:**
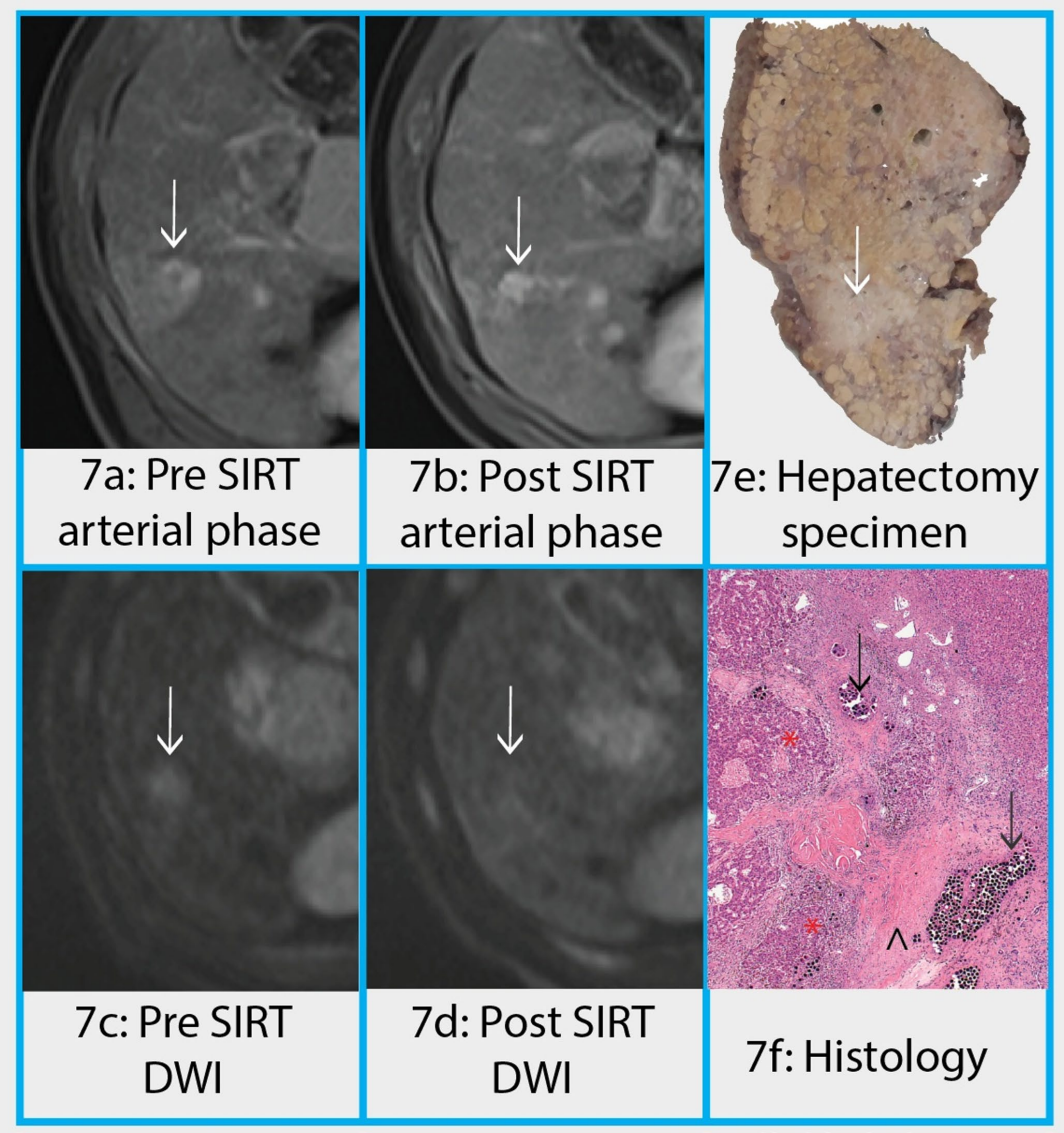
**a**–**f** Tumour (white arrow) demonstrating persistence of arterial enhancement (**a**, **b**) loss of diffusion restriction on DWI (**c**, **d**) post SIRT, with histopathology (**f**) showing 50% residual well differentiated tumour (*); fibrosis (^) and SIRT beads (black arrows)

A flowchart is presented in Fig. 3 depicting the relationship between mRECIST, DWI, and pathological outcome. MRECIST CR and loss of diffusion restriction occurred in 6 tumours; all of which had CPN (see Fig. 1 for a representative case). MRECIST CR and persistence of diffusion restriction occurred in 4 tumours, of which 3 (75%) had residual disease (representative cases Figs. 2 and 5), and 1 had complete response (Fig. 6). One case demonstrated persistence of arterial enhancement despite loss of diffusion restriction, corresponding to 50% residual well differentiated tumour on histopathological assessment (Fig. 7).

## Discussion

Evaluation of post SIRT treatment response is beset by challenges in interpretation of post radiation field changes and heterogeneity in assessment tools with limited validation. While the use of SIRT as a ‘bridge’ or ‘downstaging’ strategy for resection or transplant is increasing in popularity, overall candidate numbers remain small; the largest international series from 16 centres over 16 years produced only 100 cases with usable data [2]. Low rates of post-SIRT surgery corresponds to a low yield of pathologic specimens for the confirmation of CPN, meaning that surrogate markers such as survival metrics and longitudinal imaging are frequently used for validation of posttreatment assessment tools.

What is available in terms of pathologic-radiologic validation comes from transplant series examining loss of arterial enhancement as a predictor of complete response. A series of 37 tumours found EASL CR (equivalent to mRECIST CR) to have 100% specificity but only 52% sensitivity for pathological complete necrosis [6]. The low sensitivity for loss of arterial enhancement is consistent with another multicentre series of 33 transplants which found only 50% of tumours exhibiting mRECIST CR had pathological complete necrosis at explant [11]. Another series of 37 tumours reported a statistically significant correlation of mRECIST response to amount of tumour necrosis at histopathologic assessment however did not include specific diagnostic accuracy metrics [15].

The use of DWI in the assessment of treatment response is based on changes in water diffusivity that reflect underlying cellular alterations such as cell shrinkage and increased membrane permeability [16]. While extensively explored outside the liver, relatively few studies have examined DWI metrics after SIRT. In metastatic disease, rising ADC values have been associated with lesional shrinkage [17, 18] and similar trends have been observed post-SIRT in unresectable HCC [4, 5]. However, Vouche et al. found that both ADC and mRECIST at 1–3 months post-SIRT were poor predictors of complete pathologic necrosis [13].

In our study, we chose a qualitative visual assessment of diffusion restriction rather than quantitative ADC analysis. Although quantitative ADC assessment have been reported in similar settings in the literature, for example for treatment response in metastatic colorectal cancer [19], its reliability in the post-SIRT HCC setting remains unproven. Given that ADC values are highly sensitive to variations in scanner hardware, acquisition parameters, and motion correction, reproducibility between studies and across platforms is inherently poor. In our cohort, imaging was obtained on several scanner models and at multiple institutions, making standardised quantitative comparison impractical. Moreover, ROI-based ADC measurements are particularly vulnerable to misregistration, partial-volume effects, and post-SIRT parenchymal change, all of which compromise their biological specificity. These factors collectively undermine the assumption that numeric ADC provides an objective or standardised biomarker.

Conversely, visual comparison of diffusion restriction on serial DWI, though subjective, integrates tumour heterogeneity and local context that are often lost in averaged ADC values. This qualitative approach aligns with daily radiological practice at our institution and with the way treatment response is commonly judged in routine multidisciplinary review. A systematic head-to-head evaluation of subjective versus quantitative diffusion assessment after SIRT would indeed be valuable and remains an important topic for future investigation.

The significance of pre-treatment DWI on tumour response is unclear; in the earlier cited study of 21 HCC treated with SIRT followed by transplant, mean tumour ADC at baseline was not shown to predict response to treatment [13]. The potential of DWI as a biomarker has been summarised in a review [20] citing a number of studies that associate diffusion restriction/higher ADC values with higher histological grade/poor tumour differentiation; however the authors note that the converse relationship (i.e. non diffusion restricting/low ADC value with low histological grade/well differentiation tumour) has not been reliably demonstrated in the literature. Given our practice of non-routine pre-treatment biopsy, this relationship is difficult to ascertain and should form the subject of future enquiry.

In our study, we identified 11 tumours that did not exhibit diffusion restriction pre-SIRT (of which 6 eventually demonstrated complete response whilst five demonstrated residual disease); these were excluded from subsequent analysis of post-treatment DWI changes. The predictive ability of loss of diffusion restriction is therefore limited only to tumours that have it pre-treatment; which in our cohort remain in the majority. While underpowered to examine combined effects of both mRECIST and loss of diffusion restriction, the current study demonstrates the complementary role of DWI to augment enhancement-based assessment of response.

In keeping with findings from others [6, 13], the modest specificity (0.68) for mRECIST CR/loss of enhancement highlights the tendency to missing the diagnosis of residual disease, making it an unreliable predictor of complete response when used alone. The Li-RADS TR algorithm recently updated in 2024 to introduce DWI as an ancillary feature of viable disease and the concept of ‘mass-like enhancement’ [8] which is an advancement on response criteria of mRECIST and EASL that make no discrimination on the nature of the enhancement. Given the distortion of the tumour and tissue interface that occurs following radiation therapy, the 2024 update of Li-RADS TR in defining not simply the presence but the pattern of residual enhancement as well as formalising the role of diffusion restriction are welcome steps towards refinement of post-treatment response assessment.

In our small study, we have demonstrated the potential for persistent diffusion restriction in diagnosing residual tumour post-SIRT despite loss of arterial enhancement. However, the persistence of diffusion restriction may be due to factors other than residual tumour, such as intertumoral haemorrhage that may demonstrate resolution on interval imaging. Furthermore, the low Cohen’s Kappa of 0.197 for DWI alone (in our study partially explained by class imbalance indicated by more favourable PABAK of 0.647) means that DWI when used alone is a weak predictor of CPN and should therefore remain a complementary adjunct to mRECIST. When treated tumours demonstrate both loss of enhancement AND loss of diffusion restriction, our study suggests this combined finding might reflect a greater likelihood of CPN and should signal candidacy for a watch and wait approach, within the appropriate clinical context.

Conversely, arterial enhancement persisted despite loss of diffusion restriction in a single case where greater than 50% residual well differentiated tumour was found on histopathology, suggesting that the utility of diffusion restriction as an indicator for residual disease may in fact vary depending on tumour grade. Indeed, lower tumour grades have been shown to exhibit reduced signal intensity on DWI, which is thought to reflect similarities in the cellularity and microstructure of well differentiated lesions to surrounding background fibrotic parenchyma [21, 22].

A critical limitation of our study was the small numbers that prevented us from meaningful assessment inter-reader reliability of DWI assessments given the subjective and qualitative nature of the tool. Further limitations include the retrospective nature, which resulted in heterogeneity in time interval between SIRT-imaging-surgery. Post SIRT protocolled response imaging when done too early e.g. 1–3 months may underestimate treatment response [13]; however by assessing imaging performed closest to surgery we obtained the most representative radiologic-pathologic correlation, as well as maximised the time from treatment to transplant to ensure full treatment effect [6, 15]. Notwithstanding the challenges in coordinating post-SIRT imaging with regards to the unpredictable timing for liver transplantation; future research priorities should include prospective collection of protocolled imaging data at standardised intervals that maximise yield of post SIRT imaging/pathological changes, and collation of multi-institutional data to allow for calculation of inter-reader reliability for qualitative DWI assessment post-SIRT in order to validate its use in the broader clinical setting.

## Conclusion

In our small series, the combination of complete loss of enhancement and diffusion restriction appears to predict CPN; conversely persistence of diffusion restriction despite complete loss of enhancement was associated with residual disease in 75% of cases. While these results should be interpreted cautiously, our findings support the role of DWI as an adjunct tool for post-SIRT response assessment.

## Data Availability

No datasets were generated or analysed during the current study.
